# A Label-Free Impedimetric DNA Sensor Based on a Nanoporous SnO_2_ Film: Fabrication and Detection Performance

**DOI:** 10.3390/s150510686

**Published:** 2015-05-06

**Authors:** Minh Hai Le, Carmen Jimenez, Eric Chainet, Valerie Stambouli

**Affiliations:** 1 Université Grenoble-Alpes, CNRS, Laboratoire des Matériaux et du Génie Physique (LMGP), MINATEC, 3 parvis Louis Néel, 38016 Grenoble Cedex 1, France; E-Mails: minh-hai.le@minatec.inpg.fr or hai.leminh@hust.edu.vn (M.H.L.); carmen.jimenez@grenoble-inp.fr (C.J.); 2 School of Materials Science and Engineering, Hanoi University of Science and Technology, 1 Dai Co Viet Street, 10000 Hanoi, Vietnam; 3 Laboratoire d’Electrochimie et de Physico-chimie des Matériaux et des Interfaces (LEPMI), 1130 rue de la Piscine BP75, 38402 Saint Martin d’Hères Cedex, France; E-Mail: Eric.Chainet@lepmi.grenoble-inp.fr

**Keywords:** biosensor, DNA, label free detection, fluorescence, impedance spectroscopy, nanoporous SnO_2_ films

## Abstract

Nanoporous SnO_2_ thin films were elaborated to serve as sensing electrodes for label-free DNA detection using electrochemical impedance spectroscopy (EIS). Films were deposited by an electrodeposition process (EDP). Then the non-Faradic EIS behaviour was thoroughly investigated during some different steps of functionalization up to DNA hybridization. The results have shown a systematic decrease of the impedance upon DNA hybridization. The impedance decrease is attributed to an enhanced penetration of ionic species within the film volume. Besides, the comparison of impedance variations upon DNA hybridization between the liquid and vapour phase processes for organosilane (APTES) grafting on the nanoporous SnO_2_ films showed that vapour-phase method is more efficient. This is due to the fact that the vapour is more effective than the solution in penetrating the nanopores of the films. As a result, the DNA sensors built from vapour-treated silane layer exhibit a higher sensitivity than those produced from liquid-treated silane, in the range of tested target DNA concentration going to 10 nM. Finally, the impedance and fluorescence response signals strongly depend on the types of target DNA molecules, demonstrating a high selectivity of the process on nanoporous SnO_2_ films.

## 1. Introduction

Over the last decades, development of genosensors has increased significantly, as demonstrated by the large number of scientific publications on this topic [1]. Traditionally, DNA hybridization detection research has relied upon attachment of various labels to the molecules being studied. The common labels used in molecular biology studies to analyse DNA hybridization involve fluorescent dyes [2,3], redox active enzymes [4,5], magnetic particles [6] or different kinds of nanoparticles [7,8]. For example, the DNA target sequence is labelled with a suitable fluorescent tag. With the aid of a fluorescence microscope, fluorescence is observed at the place where complementary hybridization takes place [9]. Although these techniques are highly sensitive, label processes require extra time, expense, sample handling [10]. Additionally, labels might, in some cases, interfere with the detection, the base-pairing interaction. The challenge is to develop simple, reliable and economical methods. Label-free strategies have emerged as potential methods for detecting DNA hybridization with lower cost and high sensitivity. Label-free techniques can provide direct information on target molecules in the form of changes in a physical bulk property of a sample. Basically, label-free DNA sensors rely on the modification of a given physical parameter of the supporting material (transducer), which is induced by DNA hybridization.

Electrochemical impedance spectroscopy (EIS) has received much attention recently for the DNA hybridization detection due to its ability to perform label-free detection. EIS can sensitively detect the change of the impedance of the electrode/electrolyte interface when the DNA target is captured by the probe. EIS measurements could be performed according either faradic or non-faradic process [10]. In the case of faradic impedance spectroscopy, the addition of a redox-active species, such as [Fe(CN)_6_]^3−/4−^ [11,12] or [Ru(NH_3_)_6_]^2+/3+^ [13,14], to the bulk solution is required. Faradic EIS detection of DNA hybridization is generally based on the variation of the charge transfer resistance between the solution and the electrode surface [15]. On the other hand, no additional reagent is needed in the case of non-Faradic detection. Bio-modification of the electrode leads to the variation of either the capacitance of the double layer formed between the solution and the metal electrode surface or the capacitance located in the space charge layer at the sub-surface of semiconductive electrodes [16]. In this case, a sufficiently sensitive electrode material is strongly needed. Different kinds of sensitive materials for non-Faradic EIS DNA detection have been reported, including metals [17,18], conductive polymers [19,20,21] and semiconductors [22,23,24,25,26,27,28,29]. The latter can be divided into two categories including CMOS heterostructures [22,23,24] and single working electrodes [25,26,27,28,29].

Within this last category, our group pioneered to study the non-Faradic label-free detection of DNA hybridization based on semiconductive metal oxides as working electrodes. Dense and polycrystalline thin film electrodes constituted of CdIn_2_O_4_ [30,31] or pure/doped SnO_2_ [32,33] were elaborated by the aerosol pyrolysis technique. The detection results first showed a systematic increase of the impedance upon DNA hybridization in agreement with the field effect. In particular, we evidenced the importance of the use of non-doped films to benefit from higher field effects. Elsewhere, the high chemical stability of SnO_2_ films when dipped in saline solutions is an important criterion which led us to pursue further investigations with this metal oxide. In the following step, using an electrodeposition method, we elaborated working electrodes constituted of 1D monocrystalline nanopillars [34]. The dimensionality reduction of the SnO_2_ electrode material from 2D thin film to 1D nanopillars allowed the surface/volume ratio of the electrode to increase and thus to benefit from an enhanced field effect. As a result, the increase of the impedance signal upon DNA hybridization was more important than in the case of 2D SnO_2_ thin films. Our results showed that SnO_2_-nanopillars-electrode provides a higher sensitivity over 2D-dense SnO_2_ film electrode (97% ± 7% *vs.* 50% ± 10%) for a DNA target concentration of 2.0 µM [34]. The limit of DNA detection was found in the nanomolar range, which we expect to improve in a future study by elaborating SnO_2_ nanowires exhibiting a higher shape ratio. Presently, the idea is to reduce more the dimensionality of the electrode material down to 0D by elaborating nanoporous SnO_2_ films constituted of SnO_2_ nanoparticles and to investigate the resulting effect on the impedance signal upon DNA hybridization.

To this aim and for the first time to the best of our knowledge, in the present work, we investigated the possibility to fabricate impedimetric DNA biosensors based on nanoporous SnO_2_ electrodes. As for SnO_2_ nanopillar electrodes, the nanoporous SnO_2_ film electrodes were prepared using an electrodeposition method which provides a simpler and less expensive route to synthesize the ceramic coatings over other methods [35]. The characteristics of the obtained films, including microstructure, morphology and electrochemical properties have been thoroughly investigated using SEM, TEM and EIS. Then, a functionalization process has been carried out in order to covalently graft single strand (ss) DNA probes onto the electrode film surface. This process is based on a silanization step that we have carried out either in liquid phase or in vapour phase. EIS was used to investigate the impedance behaviour after the main steps of the functionalization process, as well as after DNA hybridization. In parallel, the DNA hybridization detection on the SnO_2_ nanoporous films was systematically checked using epifluorescence microscopy. Some performances of the sensors were also analysed, namely: sensitivity and selectivity.

The paper is organized as follows: we first present the results obtained for DNA hybridization when using the liquid phase silanization in the case of SnO_2_ films with increasing thicknesses. Then we present the results obtained when using the vapour phase silanization. The comparison between these two steps will be conducted in term of impedance variation upon DNA hybridization. Finally, the obtained results help us to have a more complete view and understanding on the effect of the SnO_2_ sensing electrode morphology and dimensionality on the response signals to non-faradic DNA detection.

## 2. Experimental Section

### 2.1. Nanoporous SnO_2_ Film Deposition

The electrodeposition of SnO_2_ thin films was carried out in a standard three-electrode electrochemical system using a computer-controlled potentiostat EG&G 322. The electrolyte consisted of 20 mM SnCl_2_·2H_2_O (>99.99%, Sigma Aldrich, MO, USA), 100 mM NaNO_3_ (>99%, Sigma Aldrich) and 75 mM HNO_3_ (>65%, Sigma Aldrich) in Nanopure water. Commercial indium tin-oxide (ITO) coated glass substrates, purchased from Advanced Film Services Company (San Jose, CA, USA) were used as the working electrodes. The thickness of the ITO layer is 300 nm, with a sheet resistance of 10 Ω/square. These substrates were sonicated in the following sequence: 15 min in ethanol, 15 min in acetone and 15 min in isopropanol in order to remove all the impurities on the surface. Then, the ITO/glass substrate was installed into the cell vertically using a specific Teflon holder which controls the area of the working electrode exposed to the electrolyte 1 cm^2^. A Pt wire and a commercial Ag/AgCl (KCl 3M) electrode were used as counter and reference electrodes, respectively. SnO_2_ films were deposited on ITO substrates at potentiostatically a fixed potential of −1.0 V (*vs.* ref.).

Cathodic electrodeposition of SnO_2_ film in nitrate solution comprises several steps [36]. First, in a strong oxidizing environment of nitric acid solution, the Sn^2+^ ions dissolved from tin dichloride are oxidized to Sn^4+^. When the negative voltage is applied, nitrate ions are electrochemically reduced at the electrode surface leading to the generation of OH^−^ by Reaction (1). These formed OH^−^ ions then reacted with the Sn^4+^ ions coming from the bulk solution to deposit SnO_2_ on the electrode surface according to Reaction (2).


NO_3_^−^ + 2H^+^ + 2e^−^ → NO_2_^−^ + 2OH^−^ (1)

Sn^4+^ + 4OH^−^ → Sn(OH)_4_ → SnO_2_ + 2H_2_O (2)

Because the total charge density (Q) is proportional to the amount of NO_3_^−^ electrochemically reduced to generate OH^−^ group at the electrode surface, Q relates to the amount of deposited SnO_2_. As the result, the film thickness could be controlled by changing the value of Q. By increasing the Q values from 0.2 to 0.8 C·cm^−2^, SnO_2_ films with increasing thickness were obtained.

### 2.2. Functionalization Process

The functionalization process of SnO_2_ films leads to a covalent attachment of DNA. It is similar to the one we previously used for SnO_2_ films and SnO_2_ nanopillars [32,34]. Briefly, it consists of the following steps: the oxide film surface was first hydroxylated using an air/O_2_ mixture plasma to create OH^−^ groups at the surface. These groups allowed covalent binding of a functional organosilane. Then a silanization step was accomplished by grafting of the 3-aminopropyltriethoxysilane (APTES). Both liquid-phase and vapour-phase procedures have been tested for APTES deposition on SnO_2_ surface:

#### 2.2.1. Liquid Phase Deposition 

The samples were located into a solution containing 0.5 M of APTES (Sigma-Aldrich) in 95% absolute ethanol and 5% deionized water under agitation for a night. To remove the unbound silane, the samples were carefully rinsed with ethanol and then, with deionized water. This process was followed by curing the samples in an oven at 110 °C for 3 h.

#### 2.2.2. Vapour Phase Deposition

The samples first were placed in a Teflon holder, which then was put into a glove bag. The next step was to draw out the air from the bag using a rotary pump and fill the bag with an argon gas. This step was repeated three times to make sure that the humidity in the bag is as low as about 5%. After 200 μL of APTES was delivered, the lid of the sample holder was closed tightly. This holder was kept at 82 °C for 1 h to cause the evaporation of APTES. To finish, the samples were rinsed carefully with absolute ethanol and deionized water to remove unreacted silane and cured in an oven at 110 °C for 1 h.

To facilitate strong covalent binding between the NH_2_ termination of APTES and the 5'-NH_2_ termination of the oligonucleotide, a cross linker molecule (10% glutaraldehyde solution in H_2_O) was applied. 20-base pre-synthesized DNA probes were used (purchased from Biomers, Ulm, Germany). A standard-type probe sequence was chosen: 5'-NH_2_-TTTTT GAT AAA CCC ACT CTA-3'. These DNA probes were diluted in a sodium phosphate solution 0.3 M/H_2_O to a concentration of 10 μM. Two μL drops of this solution were manually applied on the sample surface and incubated for 2 h at room temperature. The probes were then reduced and stabilized using a NaBH_4_ solution (0.1 M) which modifies the CH=N imine into a CH_2_-NH amine bond and also deactivates the non-bonded CHO termination of the glutaraldehyde transforming them into CH_2_-OH. The hybridization was carried out using DNA targets labeled with a Cy3 fluorescent dye. The DNA target solution was diluted in a hybridization buffer solution (NaCl: 0.5 M, PBS: 0.01 M) and spread throughout the sample surface. To minimize the experimental dilution errors, the DNA target solution was prepared once at 2 μM and was then diluted to the desired lower concentrations down to 10 nM. The samples were then placed into a hybridization chamber at 42 °C for 45 min. Finally, the samples are rinsed with saline-sodium citrate (SSC) buffer to remove all the unbound DNA targets from the surface and dried with nitrogen. In order to study the selectivity of the process, different types of DNA target have been used including complementary, non-complementary, 1- and 2-base mismatch as reported in Table 1.

**Table 1:** Sequences of the different types of DNA target.

Complementary	3' AC CTA TTT GGG TGA GAT AC-Cy3 5'
Non-complementary	3' AC TGG CGC AAT CAC TCT AC-Cy3 5'
1-base mismatch	3' AC CTA TTT G**C**G TGA GAT AC-Cy3 5'
2-base mismatch	3' AC CTA TTT G**CA** TGA GAT AC-Cy3 5'

### 2.3. Characterization Techniques

The SnO_2_ film morphology was studied using scanning electron microscopy (XL30, Philips, Eindhoven, Netherlands) and transmission electron microscopy (JEOL 2010, Tokyo, Japan). TEM and electron diffraction were carried out at 200 kV with a 0.19-nm point-to point resolution. Cross-section samples were obtained by the tripod method. Samples were polished on both sides using diamond impregnated films. Low-angle ion Ar^+^ beam milling was used for final perforation of the samples and to minimize contamination.

Impedance measurements were carried out: (I) on the bare electrodes; (II) after silanization step; (III) on the DNA probe grafted electrodes before and (IV) after DNA hybridization. The electrolyte used systematically was the pure hybridization buffer solution, containing no DNA target. A laboratory-made microfluidics cell involving a plexiglas three electrode set-up was used. In this cell, the liquid volume is 500 μL. The circular and functional surface of the film which acts as the working electrode is 0.19 cm^2^. The reference electrode is Ag/AgCl (ref.), and the counter-electrode is platinum. The electrodes are connected to a Versatile Simple Potentiostat (VSP,) impedance-analyzer (Bio-Logic, Claix, France). For EIS measurements, this apparatus is used between 10 mHz to 200 kHz with a modulation of 10 mV and an applied voltage of −0.5 V (*vs.* ref.). The impedance spectra were analyzed with Z-fit within the EC-lab software (Bio-Logic, Claix, France) using Non-linear Least Squares Fit principles.

Although this study is ultimately aimed at the development of DNA hybridization techniques which avoid the use of any label, the use of the Cy3 labelled DNA target for the impedance measurements allows the DNA hybridization validation and the systematic comparison of electrical results with the complementary optical results (fluorescence). Epifluorescence measurements were achieved using an BX41M microscope (Olympus, Tokyo, Japan), fitted with a 100 W mercury lamp, a cyanide Cy3 dichroic cube filter (excitation 550 nm, emission 580 nm) and a cooled Spot RT monochrome camera (Diagnostic, Sterling Heights, MI, USA). The Image Pro plus software (Olympus, Tokyo, Japan) was used for image analysis. The fluorescence intensity is measured at two distinct regions of the sample: the spot where DNA probes were grafted and the background outside the spot where no DNA probe was immobilized. This background intensity was then subtracted from the intensity of each spot. The fluorescence intensity value for each condition represents the average over nine different acquisitions from two independent samples.

## 3. Results and Discussion

### 3.1. Bare Electrodeposited SnO_2_ Film Characteristics

**Figure 1:**
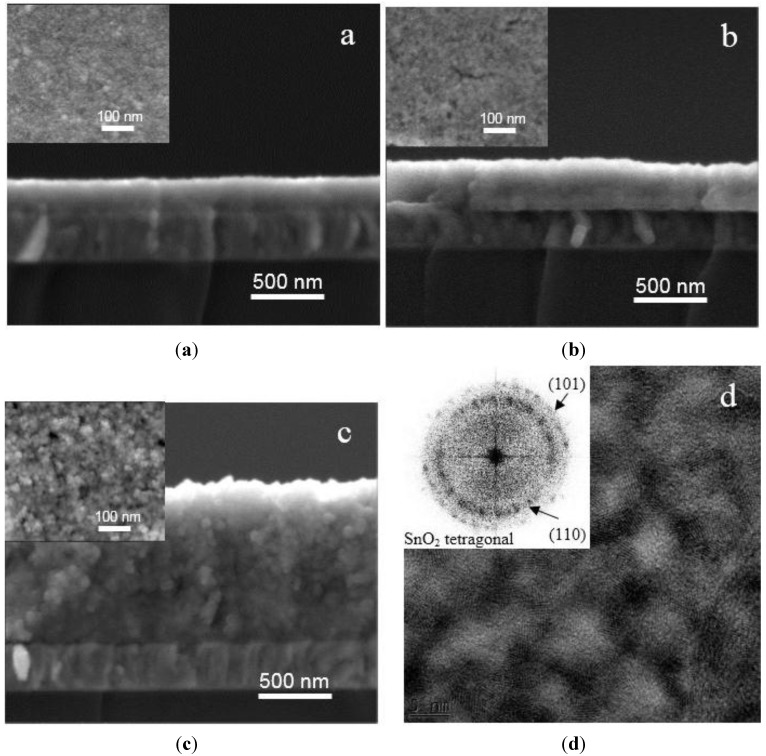
SEM images of SnO_2_ films deposited onto ITO substrate at −1.0 V (*vs.* Ag/AgCl) with charge density (Q) of (**a**) 0.2; (**b**) 0.4 and (**c**) 0.8 C·cm^−2^; (**d**) Typical cross-section HRTEM image of the film deposited with Q of 0.8 C·cm^−2^. Inset shows the corresponding SAED pattern.

The morphology of films electrodeposited with different charge densities, *i.e.*, 0.2, 0.4 and 0.8 C·cm^−2^ is revealed through typical SEM images shown in Figure 1a–c. The film thickness, determined from cross-sectional SEM images, increases linearly with the charge density. The thicknesses are 220 ± 20, 380 ± 20 and 940 ± 50 nm, corresponding to charge densities of 0.2, 0.4, and 0.8 C·cm^−2^, respectively. The top view images (inset) present a porous surface composed of numerous circular nanoparticles. The particle size does not change significantly going from 5 to 20 nm, when increasing the charge density from 0.2 to 0.8 C·cm^−2^. Due to the difficulty of observing and measuring efficiently the pore size from SEM images, the morphology of the films is further characterized by TEM observation. As expected, the cross-section bright field HRTEM micrograph reveals much better the local porous structure of the film with highly dispersed SnO_2_ nanoparticles (Figure 1d). It shows many nanocrystallites with clear lattice fringes corresponding to tetragonal SnO_2_. The average pore size is approximately 10 nm. Besides, the corresponding selected area electron diffraction (SAED) pattern (inset Figure 1d) exhibits two hollow diffraction rings corresponding to the (110) and (101) of tetragonal SnO_2_. The hollow rings reveal a quasi-amorphous microstructure of the nanoporous film which was also confirmed by grazing incidence angle XRD.

### 3.2. DNA Hybridization Detection

#### 3.2.1. Influence of Film Thickness on the DNA Hybridization Detection Signals

**Figure 2:**
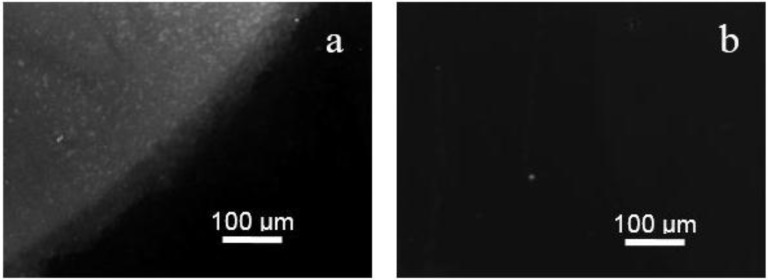
Typical epifluorescence micrographs showing the border of DNA drop on 380-nm-thick SnO_2_ nanoporous films after hybridization with Cy3 labelled targets in the case of (**a**) complementary and (**b**) non-complementary DNA hybridization with target concentration of 2 µM.

The validation of DNA hybridization on all nanoporous SnO_2_ films was first performed using epifluorescence microscopy. Figure 2a presents a typical top view image after DNA hybridization with Cy3 labelled complementary DNA targets on a 380-nm-thick SnO_2_ film. Two observations can be made. First, at the centre of the DNA drop spot, the fluorescence intensity distribution is discontinuous and discrete. Second, the border of the drop is not sharp and a fluorescence intensity gradient is observed. If these observations are similar to the ones obtained on SnO_2_ nanopillars [34], they differ from the ones obtained on dense 2D SnO_2_ thin film electrode which provided a homogeneous intensity inside the DNA drop with a sharp border [9]. The difference should be associated to the nanostructured morphology which considerably modifies and enhances the hydrophilic characteristic of the surface compared to a 2D thin film surface, causing a capillary effect and a spreading of the DNA droplet on the nanoporous surface. The fluorescence intensity significantly depends on the film thickness. The thicker the film is, the more contrasted the DNA drop is, indicating a higher amount of hybridized DNA. As a result the 940 nm thick film shows the highest fluorescence signal, *i.e.*, 1640 ± 200, while the fluorescence intensities are 270 ± 30 and 1010 ± 120 in the case of 220 nm and 380 nm thick films, respectively. To confirm that the fluorescence signal actually comes from DNA hybridization, the hybridization procedure was also carried with the hybridization solution buffer containing either no DNA target or non-complementary DNA target molecules. In the latter case, the results showed a negligible fluorescence signal coming from non-specific adsorption of DNA target (Figure 2b) while in the former case, no fluorescence signal was detected. The obtained results demonstrate the success and specificity of the used DNA hybridization process on the porous SnO_2_ films.

In a second step, the electrochemical behaviour of nanoporous SnO_2_ films with increasing thicknesses was studied (I) on the bare films and after each main step of the functionalization process: (II) after film liquid phase silanization; (III) after probe grafting (ss-DNA) and (IV) after DNA hybridization (ds-DNA). To validate that the impedance variations actually originates from DNA hybridization, impedance analyses were performed in the case of both complementary and non-complementary hybridization.

**Figure 3:**
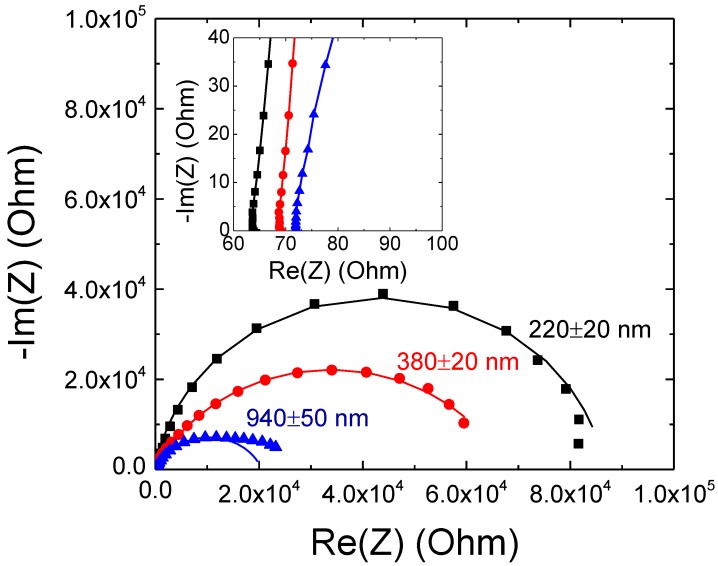
Nyquist plot (recorded at −0.5 V *vs.* ref.) of nanoporous SnO_2_/ITO electrodes with increasing film thicknesses from 220 ± 20 to 940 ± 50 nm. The filled symbols correspond to experimental data and the continuous lines to fitting data. The inset shows a zoom-in of the impedance curves at high frequency region.

Whatever the film thickness, the Nyquist plots of bare nanoporous SnO_2_ films (Figure 3) display a semi-circular shape. Besides, the semicircle diameter decreases when increasing the film thickness. The impedance of nanoporous SnO_2_ film electrode can be analysed by a simple equivalent circuit R_e_ (R_1_, Q_1_). The resistance R_e_ is the sum of ohmic resistances of both the electrolyte bulk and the electrode (ITO with SnO_2_ bulk). The parallel element circuit (Q_1_, R_1_) responsible for the observed semicircle can be essentially attributed to the polarization of the SnO_2_/electrolyte interface. Because the obtained semicircles of the Nyquist diagrams present a non-completely symmetric shape, which is due to some non-ideal behaviour, the use of a CPE instead of a capacitor is required. The impedance of a CPE is given by Z_CPE_ = (jω)^−α^/C where α is an empirical coefficient.

Extracted electrical parameters from the modelling (Table 2) showed that the resistance R_e_ increases from 64.6 to 73.9 Ω when the film thickness increases from 220 ± 20 to 940 ± 50 nm. The increase of R_e_ is mainly due to the increase of the SnO_2_ film bulk resistance with the film thickness. However, R_1_ decreases sharply when the film thickness increases. The drop of R_1_ can be explained in term of a higher real surface area in the case of the thicker films. It is believed that the increment of real surface area improves the ionic interaction at electrolyte-electrode interface resulting in low R_1_.

**Table 2:** Electrical parameter values obtained from fitting of Nyquist plot of bare nanoporous SnO_2_ electrodes with increasing film thickness.

Q (C/cm^2^)	Film Thickness (nm)	R_e_ (Ω)	C_1_ (μF)	α_1_	R_1_ (Ω)
0.2	220±20	64.61	23.37	0.779	84,782
0.4	380±20	70.40	26.29	0.772	66,496
0.8	920±50	73.90	38.15	0.810	21,276

**Figure 4:**
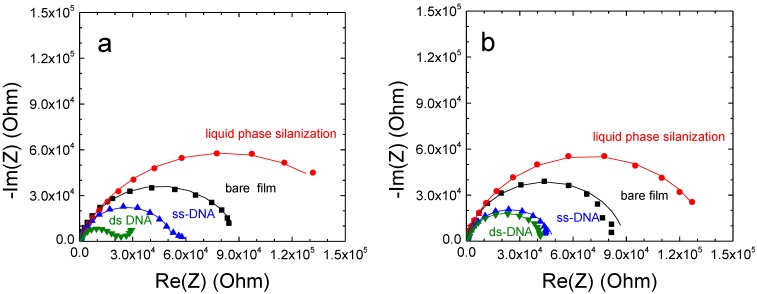
Nyquist plots of bio-modified 220 ± 20 nm thick SnO_2_ nanoporous films. DNA hybridization was performed with (**a**) complementary and (**b**) non-complementary DNA targets with target concentration of 2 µM. The filled symbols correspond to experimental data and the continuous lines to fitting data.

The overall electrochemical behaviour of bio-modified films does not change upon the functionalization step since the corresponding Nyquist plots still exhibit one large semicircle. However, their corresponding diameters undergo significantly change upon the modification step as it is shown in the case of complementary DNA hybridization for a 220 nm thick SnO_2_ film (Figure 4a) as well as in the case of non-complementary hybridization (Figure 4b). The changes are induced by the different molecular layers immobilized on the film surface. The silanization induces a large increase of the semicircle diameter, while the DNA probe grafting results in a decrease of the semicircle diameter, which is amplified upon the complementary DNA hybridization (Figure 4a). However, this last impedance change is weak in the case of non-complementary hybridization (Figure 4b). These electrochemical behaviours were systematically found for all studied films whatever the film thickness.

As for bare SnO_2_ films, all impedance curves are best fitted with an equivalent circuit R_e_(R_1_, Q_1_). In this study, we focused on the evolution of the real part of the impedance, namely the resistance R_1_. Its value could be obtained by extrapolating the fit up to the real axis. By monitoring the changes of R_1_ we can get information about the different modification steps of the SnO_2_ nanoporous based DNA sensors. We have calculated the variation of these resistances expressed as ∆R_1_/R_1_. ∆R_1_/R_1_ = [R_1_ (after hybridization) − R_1_ (before hybridization)]/R_1_ (before hybridization) × 100%. As expected, R_1_ significantly varied upon modification step. 

**Table 3:** Resistance value R_1_ obtained from fitting of the Nyquist plot of nanoporous SnO_2_ electrodes with different film thickness after liquid phase silanization, after immobilization and after complementary or non-complementary DNA hybridization with target concentration of 2 µM.

Film Thickness (nm)	DNA Target Molecules	R_1_ (Ω)				∆R_1_/R_1_ (%)
		SnO_2_ Film	Silanized	ss_DNA	ds_DNA	
220 ± 20	complementary	84782	146577	51974	24743	**−54 ± 5**
	non-complementary	87250	136078	45541	42543	**−6 ± 2**
380 ± 20	complementary	66496	115162	95137	43301	**−59 ± 5**
	non-complementary	67596	127038	101959	95300	**−5 ± 2**
940 ± 50	complementary	21276	47176	23047	15413	**−33 ± 4**
	non-complementary	20486	56258	28471	26605	**−6 ± 2**

The R_1_ values from equivalent circuit are reported in Table 3 for both films after different functionalization steps, in the case of complementary and non-complementary DNA hybridization. After silanization, the resistance R_1_ increases considerably. It could be due to the coverage of the non-charged and hydrophobic APTES layer on the electrode surface which blocks the electrolyte from diffusing within the porous layer. After ss-DNA grafting onto the silanized surface, the resistance R_1_ decreases. The negatively charged ss-DNA presumably trapped inside the nanoporous structure could facilitate the ionic current between the electrolyte and the electrode. Finally, the DNA hybridization with complementary target DNA molecules results in an additional decrease of R_1_. The decrease of R_1_ upon DNA hybridization could be explained by the observed hydrophilic character and the change of conformation linked to double-stranded ds-DNA. On the one hand, the hydrophilic ds-DNA could partially facilitate some ionic molecules of electrolyte to reach the electrode surface following their infiltration into the nanoporous structure [19]. On the other hand, the conformation of DNA changes from random coil for ss-DNA to a rigid helicoidal chain after hybridization [37,38]. Therefore, it is believed that the electrode surface could be more liberated after hybridization.

The decrease of the polarization resistance R_1_ is about −33% ± 4% for the thickest film (940 ± 50 nm) and about −54% ± 5% for the thinnest film (220 ± 20 nm). We note that whatever the film thickness, in the case of non-complementary hybridization, a weak decrease of R_1_ is obtained, *i.e.*, about 6% ± 2%. In the case of complementary hybridization, we observe that if the change of impedance is rather similar for the thin films (220 and 380 nm), it drops for the thickest film (940 nm). We attempt to explain this drop by making a relation with the percentage of surface area which is influenced by DNA hybridization. We hypothesize that due to much larger specific surface area, the amount of DNA probe and target molecules absorbed within the thicker film should be much higher than in the thin one, leading to higher fluorescence signal as mentioned above. However, because of very high specific area for the thickest film, the percentage of the surface area on which DNA probe was grafted is lower than for the thinner ones. Consequently, the DNA free surface is higher and the impedance signal becomes less important when increasing the film thickness. From this result, we deduce that the thinnest films are more relevant for observing impedance changes upon hybridization. For this reason, we follow further experimentations using the 220 nm thick films.

Besides, interesting comparisons can be made with our previous results obtained for 2D SnO_2_ dense film electrodes [32] and 1D SnO_2_ nanopillar electrodes [34]. It is to be reminded that our research work focuses on the improvement of the sensitivity performances of 2D SnO_2_ material by taking the advantage of higher developed surface of SnO_2_ nanostructured electrodes. From the results, it is clear that the sensitivity of the 0D-nanoporous film-based DNA sensors compared to that of 2D dense SnO_2_ film (−59% ± 5% *vs.* 50% ± 10%) does not improve as much as that of 1D SnO_2_-nanopillar electrode (97% ± 7%) for a DNA target concentration of 2.0 µM. However these results emphasize the importance of both the dimensional and morphological organizations of the sensing material on the impedimetric signal upon DNA hybridization. In both cases, two similar behaviours are found. First, the effect of silanization results in a large increase of the impedance, due to the non-charged APTES molecules which block the electrode surface. Second, the DNA probe grafting results in a decrease of the impedance which confirms the presence of charged molecules on both surfaces. However, we observe an opposite behaviour of the impedance upon DNA hybridization. Here it showed a decrease while it showed an increase in the case of 2D and 1D SnO_2_ electrodes. To explain this different tendency, it is to be considered that the interfacial charge distribution is different according to the electrode morphology. Regarding the 0D nanoporous films, the DNA strands and the ionic species infiltrated and are trapped within the film thickness, while they are located above the 1D nanopillars and 2D dense film surface. Generally, in the case of non-Faradic detection, DNA hybridization can induce a change of the impedance in several manners in relation with either intrinsic or external causes. 

On the one hand, in the case of 1D nanopillars and 2D dense films, the increase of the impedance upon DNA hybridization can be explained by a cause which is intrinsic to the SnO_2_ material, namely, the field effect. The addition of negatively charged DNA molecules upon hybridization leads to an increase of the space charge thickness which is located below the film surface (in the case of 2D dense film) and below the nanopillar surface. On the other hand, in the case of 0D nanoporous films, the decrease of the impedance upon DNA hybridization can be explained by some external phenomena as discussed above. The penetration of hydrophilic and charged double-stranded DNA molecules within the nanoporous film volume enhances the transport of ionic species inside the electrode volume. As a result, the impedance of this complex interface is reduced. In this case, the field effect is hindered and does not play any predominant role.

#### 3.2.2. APTES Vapour Phase Deposition *vs.* Liquid Phase Deposition

In order to obtain higher performance DNA sensor, the immobilization of the DNA probes on the film electrode needs to be well controlled. In our work, the DNA probes are covalently grafted to the aminosilane (APTES) through a cross-linker (glutaraldehyde). Functionalized surfaces were created by chemical treatment using silanization process which was first carried out in our laboratory by liquid phase deposition of a solution of silane diluted in 95% pure ethanol and 5% deionized water. However, the main issue of liquid treatment is the eventual ability of the precursor to copolymerize in the presence of water forming an inhomogeneous organosilane monolayer on the surface [39]. To overcome this problem, the vapour phase deposition has been performed in a next step. The low density of the agent in vapour phase could reduce the aggregation formation. Importantly, because the vapour is more effective than the solution in penetrating into the nanoporous structure of the films, it is expected that a superior organosilane monolayer is achieved and consequently, a better DNA surface coverage. As a result, the DNA detection performance should be enhanced.

**Figure 5:**
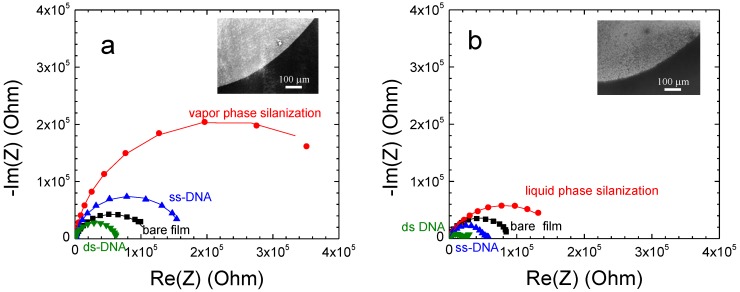
Nyquist plots of 220 nm thick bio-modified SnO_2_ nanoporous film with (**a**) vapour phase and (**b**) liquid phase silanization processes. DNA complementary hybridization was performed with target concentration of 2 µM. The filled symbols correspond to experimental data and the continuous lines to fitting data. The inset shows the corresponding typical fluorescence micrograph after hybridization of complementary target labelled with Cy3.

The Nyquist plots obtained on 220 ± 20 nm thick SnO_2_ films in the case of vapour phase silanization (Figure 5a) clearly show the importance of the silanization conditions when comparing with liquid phase (Figure 5b). In this case, the semicircle (red curve) presents a much larger diameter than that of liquid phase deposition. As previously, we perform the Nyquist plot modeling by using the equivalent circuit R_e_(R_1_, Q_1_) to determine the polarization resistance R_1_ variation upon the stepwise modification. The resistance R_1_ obtained from the impedance curve after vapour phase silanization revealed an approximately three times higher value than the one of the liquid phase silanization (432,768 Ω *vs.* 146,577 Ω in the case of liquid phase deposition). It indicates that the deposited organosilane monolayer from vapour phase was more efficient on the nanoporous film than in the case of liquid phase. DNA hybridization is then performed on both film surfaces with the same DNA target concentration of 2 μM. The change of resistance ∆R_1_/R_1_ upon DNA hybridization increases from −54% ± 5% in the case of liquid phase deposition (Table 3) to −63% ± 5% of vapour phase deposition (Table 4). 

**Table 4:** Vapour phase and liquid phase silanization: resistance value R_1_ obtained from fitting experimental data to the equivalent circuit for 220-nm-thick-nanoporous-SnO_2_ electrodes after silanization, after DNA probe immobilization and after complementary DNA hybridization with different target concentrations.

**Vapour Phase Silanization**					
**C_DNA target_ (μM)**	**R_1_ (Ω)**				**∆R_1_/R_1_ (%)**
	**SnO_2_ Film**	**Silanized**	**ss_DNA**	**ds_DNA**	
2.0	96,782	43,2768	167,202	61,836	**−63 ± 5**
1.0	86,453	370,742	147,929	76,689	**−48 ± 5**
0.5	85,310	377,590	143,687	97,006	**−33 ± 3**
0.1	81,101	416,567	121,467	99,531	**−18 ± 3**
0.01	79,987	393,879	126,847	111,929	**−11 ± 3**
**Liquid Phase Silanization**					
**C_DNA target_ (μM)**	**R_1_ (Ω)**				**∆R_1_/R_1_ (%)**
	**SnO_2_ Film**	**Silanized**	**ss_DNA**	**ds_DNA**	
1.0	97,419	144,626	47,492	34,015	**−28 ± 5**
0.5	97,926	151,935	45,305	37,707	**−17 ± 3**
0.1	95,509	135,238	43,923	40,441	**−7 ± 2**
0.01	95,264	141,849	48,337	47,538	**−2 ± 1**

This nearly 10% increase of the EIS signal confirms that the sensitivity of the DNA detection could be improved significantly by using vapour phase silanization process. The EIS result was confirmed by fluorescence measurements carried out on the corresponding samples. It showed almost three times higher fluorescence intensity in the case of vapour phase deposition over the liquid method (insets of Figure 5).

**Figure 6:**
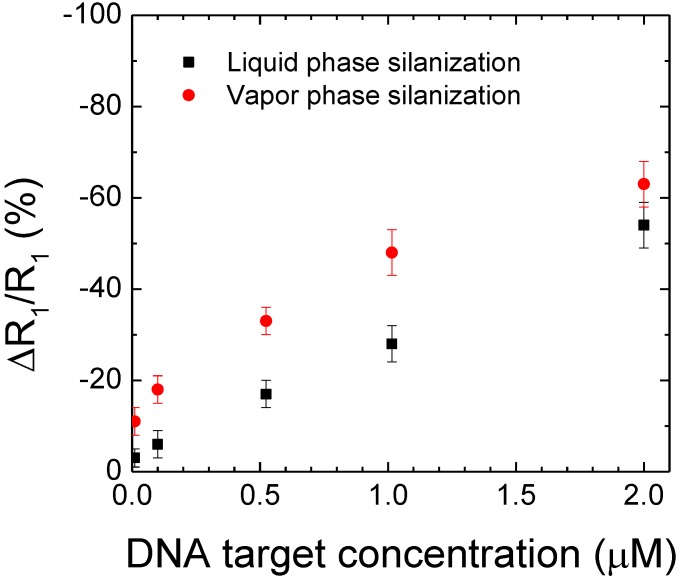
Evolution of the polarization resistance ∆R_1_/R_1_ of the biomodified SnO_2_ nanoporous films as a function of target DNA concentration in the case of vapour (red) and liquid phase silanization (black).

The sensitivity of the biomodified 220 nm thick SnO_2_ films was studied by detecting complementary DNA target at lower concentrations. The evolution of the polarization resistance ratio ∆R_1_/R_1_ has been plotted as a function of DNA target concentration (Figure 6) for both silanization processes: vapour and liquid phase deposition. The lower DNA target concentration, the less important is the decrease of resistance R_1_. In the case of vapour phase silanization, the decrease of the polarization resistance ∆R_1_/R_1_ is systematically wider, from 10% to 20%, than in the case of liquid phase silanization. Indeed, in the first case it ranges from −48% ± 5% to −11% ± 3% when decreasing DNA target concentration from 1 μm down to 10 nM (Table 4), whereas, it decreases only from −28% ± 5% to −2% ± 1% in the case of liquid phase silanization (Table 4). As expected, even for low DNA target concentrations, the infiltration of organosilane molecules into the nanopores is facilitated in the case of gas phase, which plays a role in the sensitivity enhancement of the DNA sensor.

**Figure 7:**
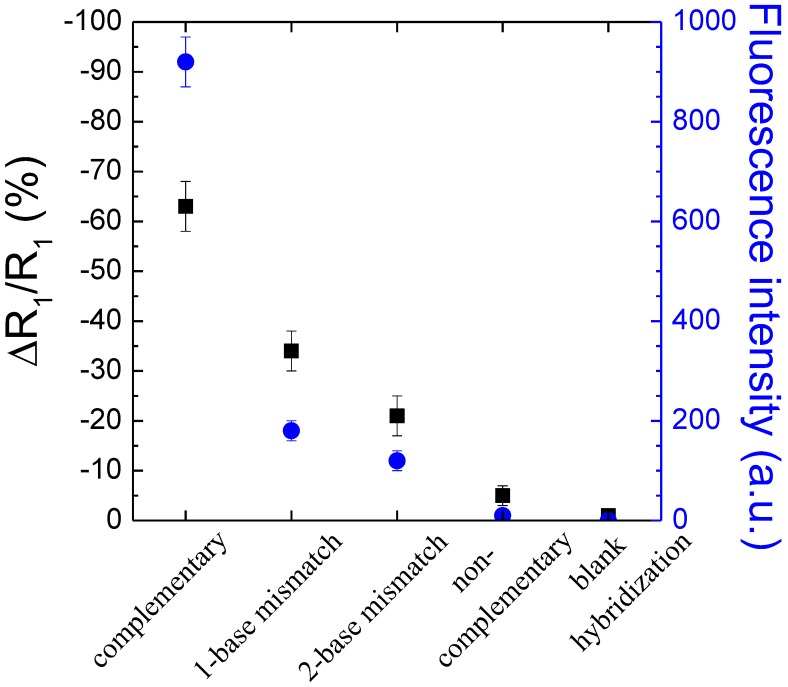
Evolutions of the polarization resistance ∆R_1_/R_1_ (%) (black) and the fluorescence signal (blue) as a function of different types of DNA target: complementary, 1-, 2-base mismatch, non-complementary and hybridization buffer without DNA target (blank hybridization).

Finally the selectivity of the sensor on the 220 nm thick nanoporous SnO_2_ based DNA sensor has further been tested by performing hybridization procedure with 1- and 2-base-mismatch DNA target molecules as well as with blank hybridization (buffer with no DNA target molecule). The concentration of all DNA targets was fixed at 2 μM. The silanization was carried out only in vapour phase deposition technique. Impedance curves exhibit one semicircles for all kinds of target molecule. The impedance curves were analysed in terms of R_1_ variations. As expected, ∆R_1_/R_1_ varies differently following the types of DNA target as can be seen from Table 5 and in Figure 7. ∆R_1_/R_1_ was equal to −34% ± 5% and −21% ± 4% in the case of 1- and 2-base-mismatch DNA targets, respectively. A negligible signal (−1% ± 0.5%) is obtained upon a blank hybridization. Such a value can be considered as background signal.

To further support the results observed in impedance measurements, the selectivity of the films has also been studied optically by epifluorescence optical microscopy. The evolutions of both fluorescence intensity and variation of resistance ∆R_1_/R_1_ respectively of the bio-modified 220 nm thick nanoporous-SnO_2_ electrode as a function of different types of DNA target are shown in Figure 7. The complementary hybridization gives the highest fluorescence signal, *i.e.*, 920 ± 70, while the fluorescence intensity significantly drops when 1- and 2-base mismatch DNA targets are used, *i.e.*, 180 ± 20 and 100 ± 20, respectively. Non-complementary hybridization provides a negligible signal, *i.e.*, 10 ± 5 of non-specific adsorption of DNA target. In the case of blank hybridization, the area where the DNA probes are immobilized could not be found. The fluorescence results matched rather well with those of impedance, which demonstrates the high selectivity of the process on nanoporous SnO_2_ sensing matrix.

**Table 5:** Vapour phase silanization: resistance value R_1_ obtained from fitting experimental data to the equivalent circuit for DNA probe immobilized 220-nm-thick-nanoporous-SnO_2_ electrodes after silanization, after DNA probe immobilization and after complementary DNA hybridization with different types of target molecules. The target concentration was fixed at 2 µM.

DNA Target Molecule	C_DNA_ _target_ (μM)	R_1_ (Ω)				∆R_1_/R_1_ (%)
		SnO_2_ Film	Silanized	ssDNA	dsDNA	
1-base mismatch	2.0	98742	356254	120292	79007	**−34 ± 5**
2-base mismatch		97249	374519	109624	86879	**−21 ± 4**
Blank hybridization (buffer without DNA target)		87876	381906	105884	99632	**−1 ± 0.5**

## 4. Conclusions

We have studied the label free DNA detection using EIS on 0D nanoporous SnO_2_ films that have been deposited by an electrodeposition process. The films thickness has been varied from 220 ± 20 to 940 ± 50 nm. The results have shown a systematic decrease of the impedance upon DNA hybridization, the decrease being more pronounced for the thinnest films. The decrease of the impedance upon DNA hybridization has been attributed to the enhanced penetration of ionic species within the film volume.

The comparison of impedance variations upon DNA hybridization between the liquid and vapour phase processes for APTES grafting on the nanoporous SnO_2_ films showed that vapour-phase method is more efficient. This is due to the fact that the vapour is more effective than the solution in penetrating into the films’ nanopores. As a result, the DNA sensors made with a vapour-treated silane layer exhibit a higher sensitivity than those produced from liquid-treated silane, in the range of tested target DNA concentrations, going to 10 nM. Finally, the impedance and fluorescence response signals strongly depend on the types of target DNA molecules, demonstrating a high selectivity of the process on nanoporous SnO_2_ films.
